# Investigating gene flow between the blind cavefish *Garra barreimiae* and its conspecific surface populations

**DOI:** 10.1038/s41598-017-05194-3

**Published:** 2017-07-11

**Authors:** Sandra Kirchner, Helmut Sattmann, Elisabeth Haring, Lukas Plan, Reginald Victor, Luise Kruckenhauser

**Affiliations:** 1 University of Vienna, Department of Integrative Zoology, Althanstraße 14, 1090 Vienna, Austria; 2 Natural History Museum Vienna, Central Research Laboratories, Burgring 7, 1010 Vienna, Austria; 3 Natural History Museum Vienna, Third Zoological Department, Burgring 7, 1010 Vienna, Austria; 4 Natural History Museum, Department for Geology & Paleontology, Burgring 7, 1010 Vienna, Austria; 5 Sultan Qaboos University, Department of Biology, Al Khoudh, Muscat, 123 Oman

## Abstract

Cave-dwelling taxa often share the same phenotypic modifications like absence of eyes and pigmentation. These “troglomorphic characters” are expressed in the populations of *Garra barreimiae* from the Al Hoota Cave and nearby Hoti Pit in Northern Oman. Surface morphotypes of this cyprinid species are common throughout the distribution area. Very rarely individuals with intermediate phenotypes can be found. In the present study, potential gene flow between cave and surface populations was tested and population structure within five sampling sites was assessed. Overall, 213 individuals were genotyped at 18 microsatellite loci. We found that the cave populations have lower genetic diversity and are clearly isolated from the surface populations, which seem to be sporadically in contact with each other. The results indicate a recent genetic bottleneck in the cave populations. Thus, it can be assumed that during climatic changes the connection between cave and surface water bodies was disjoined, leaving a subpopulation trapped inside. Nevertheless, occasional gene flow between the morphotypes is detectable, but hybridisation seems only possible in cave habitat with permanent connection to surface water. Individuals from surface sites bearing intermediate phenotypes but cave genotypes imply that phenotypic plasticity might play a role in the development of the phenotype.

## Introduction

Troglomorphic cave fish always share a similar phenotype exhibiting reduction or absence of eyes and pigmentation. These “typical” cave characters developed independently among taxa and can vary strongly in their expression, often even within species or populations^1^. The best studied organism concerning this matter is the characid *Astyanax mexicanus* (Filippi, 1853)^2–10^. Besides *Astyanax*, the Afro-Asian cyprinid genus *Garra*, which comprises over 100 freshwater fish species can be considered as an exemplary system. It includes several cave-dwelling species or populations within species exhibiting varying degrees of troglomorphism. A mental disc (modified lower lip used to adhere to the substrate) is characteristic to most species of the strictly continental genus which is supposed to have originated in the early Miocene around 23 Myr in Southwestern China^11–13^. Typical troglomorphic characters as complete lack of pigmentation and complete anophthalmy are displayed in the Iranian cave barb *Garra typhlops* (Bruun & Kaiser, 1944)^14^ and in the Iraqui blind barb *Garra widdowsoni* (Trewavas, 1955)^15^, both species being cave-dwelling without any surface occurrences known. This is also the case for the Somalian cave-dwelling species *Phreatichthys andruzzii* Vinciguerra, 1924, for which Colli and colleagues^16^ suggested a taxonomic update due to the position of *P*. *andruzzii* within the genus *Garra* in their phylogenetic tree. This species, which according to Calderoni and colleagues^17^ evolved in complete darkness for at least 2.8 million years, exhibits also pronounced troglomorphic traits^18^.

In the centre of the present study is *Garra barreimiae* FOWLER & STEINITZ, 1956, which, like *A*. *mexicanus* comprises both, surface and cave dwelling populations. *Garra barreimiae* is endemic to the southeastern Arabian Peninsula. It is quite common in the United Arab Emirates and northern Oman along the Hajar Mountains where it inhabits all kinds of water puddles, streams and even artificial water drainage systems (aflaj/falaj) used by the locals to water their gardens.

In 1980 Dunsire and Gallagher discovered a subterranean population of *G*. *barreimiae* in the Al Hoota Cave near the Jebel Akhdar in northern Oman. Due to the morphological similarities, it was assigned to the species *G*. *barreimiae* by Banister^19^. Yet, the individuals of this population exhibit a typical troglomorphic phenotype such as no pigmentation and lack of externally visible eyes, while scales are present. Besides the more obvious differences, Banister also described the troglomorphic specimens to exhibit smaller optic lobes and reduced squamation. In the closely located cave Hoti Pit^20^, we discovered another population of *G*. *barreimiae* which also displayed the cave morphotype.

Kruckenhauser and colleagues^21^ were the first to conduct molecular genetic analyses in this species. They sequenced a part of the mitochondrial (mt) control region (CR) in individuals from both the Al Hoota Cave population as well as from several surface sampling sites. Their results showed that all individuals collected in the cave belong to the same haplogroup. However, this haplogroup also contained individuals from nearby surface localities. Most of the latter individuals exhibited an intermediate phenotype suggesting that the cave and surface populations might be still in contact or have been until recently. The genetic distances between individuals from Al Hoota Cave and individuals from nearby surface localities are quite small implying a recent origin of the cave population. Yet, the question remained whether a nuclear marker would confirm the close phylogenetic relationships between surface and cave populations as suggested by the mt CR. Within the genus *Garra*, *G*. *barreimiae* represents the rare case where both morphotypes (troglomorphic/surface) are still existing within one species. The aim of the present study was to better understand this system in which both forms co-exist. More specifically, we asked, if there is recent gene flow between those populations and whether it does occur equally in both directions? Are the individuals exhibiting an intermediate phenotype the result of recent hybridization between cave and surface individuals or a result of phenotypic plasticity? What is the genetic structure and diversity of cave and surface populations of *G*. *barreimiae*? Do the nuclear markers confirm the presumed bottleneck events as suggested by mt data?

To address those questions we utilized 18 of the 19 microsatellite loci designed for *G*. *barreimiae* by a 454 sequencing approach^22^ and analysed them in Al Hoota Cave as well as several surface populations in Northern Oman. Moreover, morphological measurements of the eye size were taken to assess the amount of reduction. The results were compared with those derived from mt CR sequences.

## Material and Methods

### Sampling

Tissue samples (finclips) were taken from a total of 213 individuals collected at five sampling sites located at the south side of Central Hajar Mountain chains in northern Oman, some 25 km northwest of the town Nizwa. These specimens were captured via fishing net and immediately anesthetized with clove oil and stored in 80% ethanol. Two of the sampling sites are mere cave locations, two are typical surface sites and one location consists of subterranean water bodies connected to surface water bodies (for more detail see Supplementary Figure S1):

1. The Al Hoota Cave (AH, also Khaf Hoti) is a 5 km long spacious cave southeast of the Jebel Shams mountain peak (2980 m above sea level (asl)) that developed in cretaceous limestone. The cave forms an underground flood system: during flood events water from the above lying Wadi Hoti sinks into the upper entrance of the cave (990 m asl) and re-emerges 262 m deeper at the lower entrance into the Wadi Falahi^20^. These floods occur occasionally every few years or sometimes more than once a year. Between such events there is almost no running water in the cave, but there are four extensive lakes and some smaller ones. These water bodies hold only troglomorphic fish^20^. Between the lakes there are several vertical drops that seem to be an insurmountable barrier for fish (from lower to higher lakes). From the passage morphology and transported cave sediment it can be estimated that during floods flow velocities in some parts reach more than 10 m/s and discharge rises up to few hundred litres per second. The average temperature in the cave is 25 °C and humidity between 40–70% up to 100% after floods when the water in the lakes is high and sumps stop air ventilation^23^. Except for floods, the wadis up- and downstream of the cave are dry. The lower entrance of the cave was developed as a show cave. Geographic coordinates: longitude 57.3572; latitude 23.0844.
2. Hoti Pit (HP) is a pitfall in Wadi Hota 400 m upstream and some 20 m higher than the upper entrance to Al Hoota Cave. During floods Hoti Pit takes some water from the Wadi Hoti but according to sediments and morphology only few m^3^/s. The entrance is a 12 m deep pit and in the lake at its bottom no fish were found. The known end of the 380 m long cave is formed by another lake harbouring only troglomorphic fish. The part of the cave where the passage ceiling reaches into the water and forms a sump, has not been explored yet and the reappearance of the water is unknown. As there are no signs of backflooding at the terminal sump it can be concluded that the cave behind the sump is spacious. Geographic coordinates: longitude 57.3726; latitude 23.1015.
3. Ghubrat Tanuf (GT) is a cave located in the Wadi Falahi at 670 m asl and approx. 1 km downstream of the lower Al Hoota Cave entrance. A small stream emerges from the cave entrance that feeds a pool in the Wadi (called Ain Al Msalla). The rather narrow cave is an almost horizontal water table cave with a length of 690 m. Estimated discharge values reach from less than 1 l/s to few l/s. During floods, water from the Wadi enters the cave for some 100 m as indicated by wood and garbage transported into the cave. In the upstream end the cave splits up into two separate streams which both emerge from sumps. The upper stream is interrupted by a 9 m high cascade where no fish were found in the upstream part. The lower one is only marginally higher than the entrance, and surface fish can be found throughout this part. In this cave, mostly individuals displaying the surface morphotype occur, but also few individuals exhibiting typical cave or intermediate phenotypes were found. Geographic coordinates: longitude 57.3681; latitude 23.0718.
4. The sampling site Wadi Falahi (WF) is a surface water body about 200 m underneath the lower Al Hoota Cave entry which represents the beginning of the Wadi Falahi, a riverbed which reaches ~4.5 km downstream comprising also the sampling site Ghubrat Tanuf/Ain al Msalla. Most of the time, the wadi lies dry, but single puddles may remain for several weeks or even months after rains. Fish were collected at a rather shallow water body located about 680 m asl which appears to be drying out regularly during summer. At this sampling site, usually the surface form occurs but also few individuals exhibiting intermediate phenotype were found. Geographic coordinates: longitude 57.3539; latitude 23.0804.
5. Misfat al Abriyeen (abbr. Misfat, MF) is a small mountain village located at an impressive wadi at 1000 m asl about 7 km northwest of Al Hoota Cave. At the end of (0.7 km behind) the village a spring emerges from big boulders in the wadi and is tapped into an artificial water drainage system (aflaj). All *G*. *barreimiae* from Misfat al Abriyeen had the surface phenotype and all specimens analysed were collected from this aflaj. Geographic coordinates: longitude 57.3159; latitude 23.1481.

In Summary, within the two enclosed caves (Al Hoota Cave and Hoti Pit), only the troglomorphic form was found. The water body of Ghubrat Tanuf cave is constantly connected to the surface water body of Ain Al Msalla and harbours mostly fish bearing a typical surface phenotype, but also few individuals with intermediate or troglomorphic phenotype. A similar phenotypic composition also applies for the surface water body Wadi Falahi, whereas in Misfat only fish with the surface phenotype were found.

### DNA extraction, PCR amplification and genotyping

We extracted genomic DNA from tissue (finclips preserved in 80% ethanol) following the QIAGEN DNeasy Blood & Tissue extraction kit protocol. All samples were profiled with 19 microsatellite loci by using primers that were specifically designed for *G*. *barreimiae* ^22^. One microsatellite marker published in ref. 22 turned out to be working improperly (~35% missing values over the whole dataset) and was therefore excluded from the data analyses. Thus, the comprehensive analysis was performed with 18 loci. In the current study, we applied the multiplex amplification protocol established in our previous study using also the same set of labelled primers. The microsatellite amplification products were visualized on a 3130xl Sequence Analyzer (Applied Biosystems, USA). The FASTA files delivered by the sequencer were imported into the program GeneMapper version 5.0 (Applied Biosystems, USA) and analysed using adapted settings for automatic allele recognition (bins). All allele calls were also checked manually to avoid misinterpretation of noise data or artefacts and to review ambiguous alleles. Amplifications that were too weak to resolve the peaks or which had any extra peaks were re-amplified and rerun up to four times. Finally, any remaining unresolved alleles were treated as missing data. A list containing the reviewed genotypes of all individuals analysed was used for data analysis (Supplementary Table S1). In this manuscript, the term “genotype” is used to describe the microsatellite allele composition characteristic for a population or morphotype.

In 96 selected samples, we amplified a section of the mt CR gene via PCR reaction (25 µl reaction volume: 1 µl DNA, 5 µl Q-solution, 2.5 µl buffer, 500 µM dNTPs, 1.5 µl (1.5 mM) Mg2+, 0.25 µl of each primer (50 pmol/µl of both primers, 0.1 µl TopTaq-polymerase (0.5 units), 13.9 µl a.d.). The following primers were used: Thr1+ 5′-GCATCGGTCTTGTAATCCGA-3′, and CR4- 5′-TTGGGCGTCGGCGGTGAGAG-3′; designed by ref. 21). The cycling protocol was: initial denaturation at 94 °C for 4 min, 35 cycles denaturation at 94 °C for 30 s, annealing at 58 °C for 30 s, extension at 72 °C for 40 s, and final extension at 72 °C for 7 min. The resulting PCR products were purified and sequenced in both directions at LGC genomics (Berlin, Germany) using the PCR primers. The obtained sequences were edited in BioEdit version 7.2.5^24^, and aligned with the program MEGA version 6.06^25^ together with the 122 sequences retrieved from Kruckenhauser *et al*.^21^. The final alignment was 399 bp long and comprised a total of 218 sequences from nine sampling sites. The haplotype network was constructed using the software PopART version 1.7 (available at http://popart.otago.ac.nz) and the Median-joining (MJ) algorithm proposed by Bandelt, Forster and Röhl^26^.

### Data analysis

#### Genetic diversity

We explored the data mostly by employing the statistical computing software environment *R* version 3.1.3^27^. After checking our data set for completeness and missing values (0.57% mean missing data within the whole data set), we calculated general information like the number of alleles, the diversity index and evenness per locus with the *R-package poppr* version 2.1.0^28^. Furthermore, we calculated the expected (H_e_) and observed (H_o_) heterozygosity per locus in each population with the *R-package adegenet* version 2.0.0^29^ and the mean H_e_ and H_o_ over all loci per population with the *R-package diveRsity* version 1.9.89^30^. In addition, we tested for genotypic linkage disequilibrium with the software *Genepop* version 4.3^31^. Deviations from the Hardy-Weinberg equilibrium (HWE) were estimated using both the global test and exact test in *Genepop* executing 10000 Markov chain Monte Carlo (MCMC) runs for 100 batches, each with 5000 iterations. The software *Micro-Checker* v. 2.2.3^32^ was used to check for the existence of microsatellite null alleles in the data set. Although there was only one population, i.e., Hoti Pit, with no signal of microsatellite null alleles at any locus, the tests showed that microsatellite null alleles at a low frequency might be present in populations Ghubrat Tanuf (6 loci showing signs of microsatellite null alleles) and Wadi Falahi (7 loci showing signs of microsatellite null alleles), whereas in the populations Al Hoota and Misfat potential microsatellite null alleles are only present at one locus. However, since none of these loci showed a constant significant deviation in all populations, and further analysis indicated population admixture between Ghubrat Tanuf and Wadi Falahi (see below) we did not exclude these loci from our analyses. We tested the data set also for linkage disequilibrium and the results showed that some locus pairs exhibit evidence of linkage disequilibrium in single populations, but never in all populations.

In order to statistically describe the level of inbreeding or differentiation among and within populations, we calculated the fixation indices as implemented in the *R-package pegas* version 0.8.2^33^. This function computes F_it_, F_st_ and F_is_ for each locus in the data set according to the formulae of Weir and Cockerham^34^. Subsequently, we also calculated the pairwise Fst-values between all populations with the *Microsoft Excel Add-In GenAlEx version 6*.*5* ^35, 36^. The inbreeding coefficient F_is_ after Weir and Cockerham over all loci per population was calculated with the program *FSTAT* ^37^.

Allelic richness per locus per population was computed to take the differing sampling sizes of populations into account. This was done by using 1000 re-samples of the smallest sample in the data set (Hoti Pit with N = 17) via the *R-package diveRsity*. Private alleles were estimated with the *R-package poppr* and the percentage of private alleles per population was calculated based on the actual amount of alleles per population.

#### Population Structure Analysis and Differentiation

The software *structure* ^38^ was used to perform population structure analyses. We employed the admixture model and tested for clusters from K = 1 to K = 7 for the whole data set and K = 1 to K = 3 for the cave subset, and for each assumed K 20 iterations per run were performed to find the correct number of clusters (K). Length of burnin was set to 100000 and the number of MCMC repeats after burnin was 500000. The results were then revised with the online program *structure Harvester* web version 0.6.94^39^ to explore which K has the highest probability and ΔK. The next step was to summarize the iterations and to minimize the variance across all twenty iterations of the selected ΔK value with the software *CLUMPP* version 1.1.2^40^. For high quality visualization of the combined results obtained via *CLUMPP*, we used the software *Distruct* version 1.1^41^.

A principle component analysis using the *R-package ade4* version 1.7–2^42^ was performed. By using the *scaleGen* function in the *R-package adegenet*, which replaces the missing values with generated mean allele values, it was possible to include all individuals.

To infer whether gene flow is recent we used the program GeneClass2^43^ for real-time estimation of first-generation migrants. We used the L_home likelihood computation and the Bayesian method criterion^44^ and applied the simulation algorithm suggested by Paetkau *et al*.^45^. The calculation was conducted with a number of 10000 simulated individuals and Threshold was set on 0.05.

#### Morphological Eye Measurements

We measured eye diameter of intermediate individuals which were collected at the sampling sites Ghubrat Tanuf (N = 11) and Wadi Falahi (N = 14). As not all specimens were fully grown adults, we scaled the eye diameter as percentage proportional to the head length (from snout tip to end of operculum) to allow comparison between individuals of different sizes. Subsequently, we also measured eye diameter of surface individuals with external fully developed eyes and pigmentation (N = 9, three specimens of each surface site - Ghubrat Tanuf, Wadi Falahi and Misfat Al Abriyeen). The according measurements﻿ and photographs are provided in Supplementary Table S3 and Supplementary Figure S2.

## Results

### Genetic diversity within sampling locations

**Figure 1:**
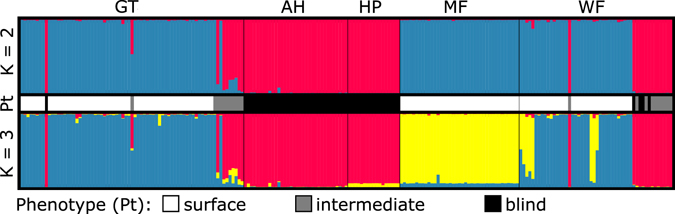
Population structure analysis based on the complete data set. The two colours in the top section (K = 2) represent the two most probable clusters (blue = surface cluster, magenta = cave cluster), whereas the bottom section shows the population structure analysis with the second highest probability (K = 3). The three colours represent two surface clusters (blue and yellow) and a cave cluster (magenta). The bar between the two sections resembles the phenotype (Pt) of the individuals analysed: white = surface, grey = intermediate, black = blind.

At first, in order to assess the genetic variation among and between specimens from different sampling sites in general, we calculated the descriptive statistics (Table 1) for the 18 unlinked microsatellite loci. All loci were polymorphic over the whole data set (between 2 and 23 alleles per locus). Concerning single sampling locations, there were a few monomorphic markers in each. In Ghubrat Tanuf the range of allele number per locus ranged from 1 (3Z7I) to 21, in Al Hoota from 1 (3Z7I, 3N43, 2PUM) to 14, in Hoti Pit from 1 (QLIM, JQSO, 3Z7I, 3N43, CB75, 2PUM) to 8, in Misfat from 1 (QLIM, 3N43) to 10 and in Wadi Falahi from 1 (3Z7I) to 18. None of these markers were monomorphic in all populations hence we did not exclude them from the data set. We repeated all descriptive statistic calculations with the data set divided into groups of morphotypes (surface/cave) according to the results of the structure analysis (see Fig. 1) disregarding the geographic origin (population) of the individuals (data shown in Supplementary Table S2).

**Table 1:** Sample information and population genetic parameters of the five populations investigated.

Population	N	Na	A	Ar	pA	pA%	H_o_	H_e_	F_is_	p	Ld	Lp%	HWE
GT	73	119	6.61	4.91	10	8.40	0.48	0.56	0.14	0.24	PH8A*, UHPE*, FYP9*, I6G2*, 3ROZ*	94.44	0.000
AH	34	68	3.78	3.08	8	11.76	0.37	0.37	0.03	0.64	UHPE*	83.33	0.374
HP	17	41	2.28	2.15	1	2.44	0.26	0.25	−0.01	0.23		66.67	0.522
MF	39	77	4.28	3.68	9	11.69	0.49	0.50	0.04	0.43	CJHG*, 9XNC	88.89	0.027
WF	50	123	6.83	5.29	12	9.76	0.50	0.62	0.21	0.19	JQSO*, CJHG*, 88CM*, I6G2*, 3ROZ*, 2PUM*	94.44	0.000

N Number of analysed individuals, Na number of alleles per population, A mean number of alleles per locus, Ar allelic richness, pA number of private alleles, pA% percentage of private alleles per population based on the actual amount of alleles per population, H_o_ mean observed heterozygosity over all loci, H_e_ mean expected heterozygosity over all loci, F_is_ inbreeding coefficient, p (mean) p-value for Hardy-Weinberg equilibrium (global test, mean over all loci), Ld Loci, that deviated from HW-equilibrium (loci with potential microsatellite null alleles are marked with an asterisk), Lp% percentage of polymorphic loci, HWE p-value for Hardy-Weinberg equilibria (global test).

The number of alleles (surface: 119 in Ghubrat Tanuf; 77 in Misfat, 123 in Wadi Falahi; cave: 68 in Al Hoota; 41 in Hoti Pit) and the proportion of polymorphic loci (surface: 17 in Ghubrat Tanuf, Misfat and Wadi Falahi; cave: 14 in Al Hoota, 12 in Hoti Pit) were generally higher in surface than in cave populations. Regarding the genetic variability, we corrected these results by taking the variable sampling sizes into account and calculated the allelic richness according to the sample with the fewest individuals (Hoti Pit with 17 individuals). The average allelic number turned out to be slightly lower in the cave populations (3.08 in Al Hoota, 2.15 in Hoti Pit) than in the surface populations (4.91 in Ghubrat Tanuf, 3.68 in Misfat, 5.29 in Wadi Falahi). Furthermore, the calculated mean H_e_ over all loci was higher in surface populations (55.77% in Ghubrat Tanuf, 50.09% in Misfat, 62.29% in Wadi Falahi) compared to the cave populations (37.45% in Al Hoota, 25.31% in Hoti Pit). In the populations Ghubrat Tanuf and Wadi Falahi H_e_ was higher than H_o_, whereas both parameters were similar in the other populations. The most private alleles were found in the Al Hoota and Misfat populations (proportion of private alleles: 11.76% and 11.69%, respectively). In the populations Ghubrat Tanuf and Wadi Falahi, less private alleles were detected (8.40% and 9.76%), whereas the fewest private alleles were found in the Hoti Pit population (2.44%).

Deviations from Hardy-Weinberg equilibrium (HWE) for each population and locus showed that the surface populations exhibit more loci with significant deviation (5% level) than the cave populations (surface: 6 Wadi Falahi, 4 Ghubrat Tanuf, 2 Misfat; cave: 1 Al Hoota, 0 Hoti Pit). The results from the global HW test indicate that the Ghubrat Tanuf, Misfat and Wadi Falahi populations are not in HW equilibrium, whereas the two cave populations Al Hoota and Hoti Pit showed no significant deviation for HW equilibrium. These results remained after repeating the global HW test excluding individuals with an intermediate phenotype (data not shown). The reason for deviations from HWE in the surface sites is most likely due to the presence of microsatellite null alleles. The few loci showing deviations from HWE are the same for which potential microsatellite null alleles were detected. Another reason could be occasional migration from one surface population to another (indicated in Fig. 1), which is a violation of the assumptions for testing HWE^46^.

The inbreeding coefficient F_is_ is positive in all but the Hoti Pit populations indicating at least some degree of heterozygote deficiency. However, these F_is_-values are close to zero (which would indicate complete panmixia) in Hoti Pit, Al Hoota and Misfat, while the highest values were found again in Ghubrat Tanuf (0.14) and Wadi Falahi (0.21).

### Genetic differentiation between sampling localities and potential gene flow between cave and surface populations

The next step was to learn more about the genetic population structure and to test, if groups can be detected and whether these groupings are in accordance with the sampling sites and/or phenotypes. In the *structure* analysis K = 2 yielded the highest probability, and also the ΔK was highest for K = 2 (Fig. 1). In fact, the clear discrimination here lies between the populations from Ghubrat Tanuf, Misfat and Wadi Falahi (surface or constant contact to surface) on the one hand and the enclosed cave populations of Al Hoota and Hoti Pit. The latter seem to be strongly isolated from the surface populations. Nonetheless, the situation in the surface cluster is more complex: some individuals exhibit an intermediate phenotype. They belong genetically either to the cave cluster or they represent an intermediate genotype. These intermediate phenotypes are only found in Ghubrat Tanuf and Wadi Falahi. Of these two localities, only Ghubrat Tanuf has contact to underground water bodies.

When using the second highest probability at K = 3, further structuring can be found within the surface cluster. The Misfat population is differentiated from Ghubrat Tanuf and Wadi Falahi populations, although some individuals belonging genetically to Misfat can also be found in the Wadi Falahi cluster (Fig. 1).

In respect to the result of the *structure* analyses (K = 2), we split the data set accordingly into two groups, a cave cluster including the Al Hoota and Hoti Pit individuals and a surface cluster comprising individuals from Ghubrat Tanuf, Misfat and Wadi Falahi.

**Figure 2:**
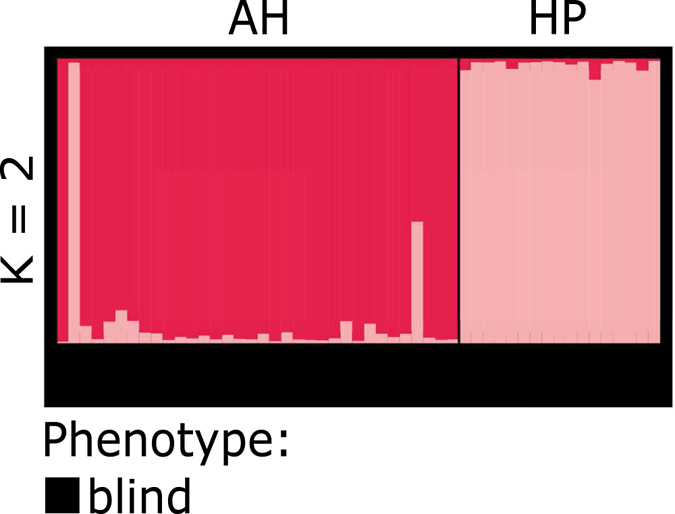
Population structure analysis based on the cave data set. The figure (K = 2) represents the two most probable clusters (magenta = Al Hoota, light pink = Hoti Pit) resulting from the structure analysis. All individuals belonging to this data set bear a cave phenotype (indicated by the bar below).

**Figure 3:**
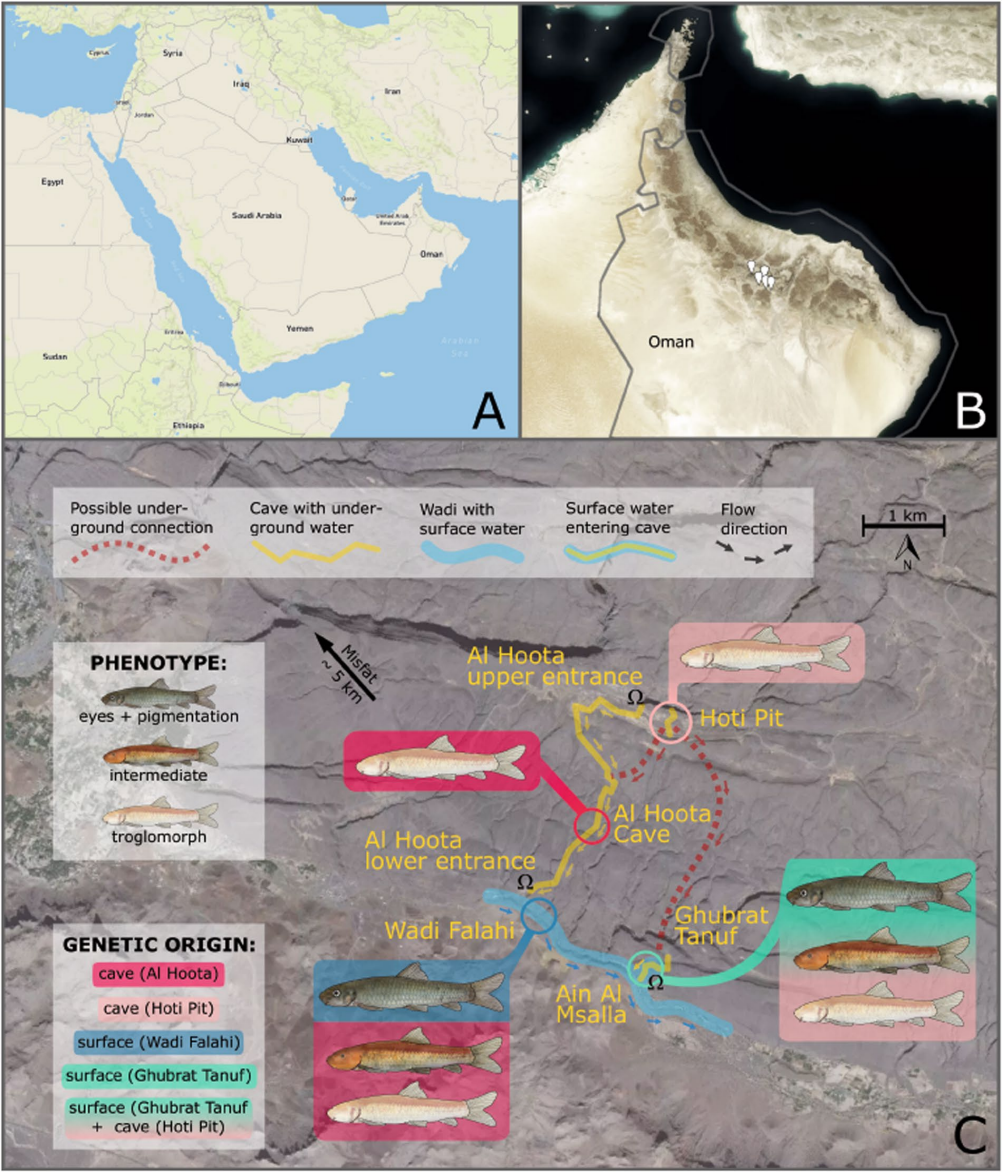
Geographic map of sampling area in detail. The figure shows a detailed map of the sampling area in northern Oman in the midst of the Hajar Mountains pointing out the sampling sites and showing the system in general (excluding Misfat). Information on which phenotypes were found at the sampling sites and their genetic assignment is provided. Caves with underground water bodies are displayed with a yellow line, the wadi (i.e., surface water bodies) is marked with a blue line and possible subterranean connections are indicated with the dotted red line. The contact zone between underground and surface water (passage from Ain al Msalla to Ghubrat Tanuf) is marked with a yellow line. Arrows indicate flowing direction of the water stream. The map was created with the program TileMill version 0.10.1 (https://tilemill-project.github.io/tilemill/).

Besides, we also performed a *structure* analyses with a subset of individuals exclusively collected at the two cave sites (Al Hoota, Hoti Pit) to explore whether they are genetically homogenous or if they are differentiated from each other. The analysis resulted in the highest probability of K = 2, with the two clusters correlating with the two sampling sites (Fig. 2). Interestingly, the Al Hoota population contains also two individuals bearing at least part of the Hoti Pit genotype whereas no Al Hoota genotype was found in the Hoti Pit population. A summary of the geographic position, possible connections and the according phenotype and genotype affiliation of the individuals analysed at each sampling site is provided in Fig. 3.

**Figure 4:**
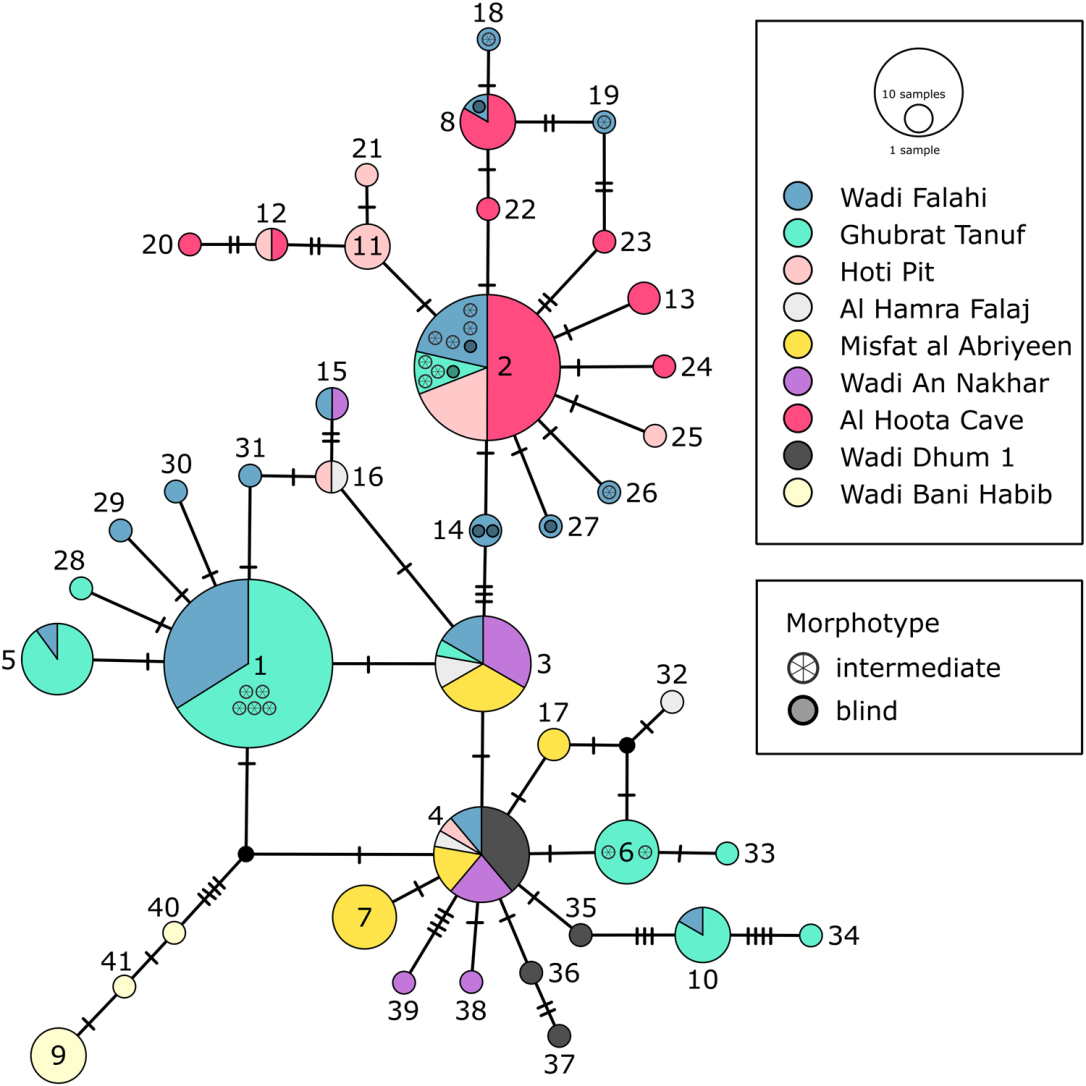
Median-Joining network based on CR sequences. Each circle represents a haplotype and the sizes of circles correspond to the number of individuals sharing this haplotype. Colours indicate sampling site. Every crossbeam on the connecting lines between haplotypes represents a substitution. Small black dots between haplotypes symbolize hypothetic haplotypes not present in the data set. Blind individuals from either Ghubrat Tanuf or Wadi Falahi are indicated by shaded circles, intermediate individuals are indicated by circles filled with an asterisk.

A MJ network based on a 399 bp long fragment of the CR gene is shown in Fig. 4. The GenBank accessi﻿on numbers for the 96 CR sequences (MF183116-MF183211) are listed in the Supplementary Table S4. The network consists of 41 haplotypes, of which five are rather frequently occurring in ten or more individuals (HT 1 to 5), twelve haplotypes occur in two to eight individuals (HT 6 to 17) and 24 haplotypes are represented by only one individual (HT 18 to 41). All samples of troglomorphic cave fish are grouped in a “cave” haplogroup (HT 2, 8, 11 to 13, 20 to 25) which also contains haplotypes of all intermediate/blind individuals from Wadi Falahi (HT 2, 8, 18, 19, 26, 27) as well as three intermediate and one blind individual from Ghubrat Tanuf (HT 2). Not a single surface phenotype individual is present in this haplogroup. The remaining intermediate individuals from Ghubrat Tanuf appear together with surface individuals from either Ghubrat Tanuf of Wadi Falahi (HT 1, 6). This outcome generally supports the findings of Kruckenhauser *et al*.^21^, but contains also samples from Hoti Pit, more samples of intermediate individuals and additional samples from the other sampling sites (see also Supplementary Table S4).

The fixation indices (Fst) and pairwise Fst-values (Nei’s unbiased genetic distance) between all pairs of populations were calculated (heterozygosities are weighted by group sizes). The values range from 0.296 (Misfat and Hoti Pit) to 0.020 (Ghubrat Tanuf and Wadi Falahi) (Table 2).

**Table 2:** Fst-values between all pairs of populations calculated with GenAlEx.

Fst	GT	AH	HP	MF
AH	0.172***			
HP	0.221***	0.103***		
MF	0.087***	0.223***	0.296***	
WF	0.020***	0.109***	0.159***	0.077***

Fst fixation indices of the five populations investigated: GT Ghubrat Tanuf, AH Al Hoota Cave, HP Hoti Pit, MF Misfat, WF Wadi Falahi. P-values indicated by asterisk: ***< = 0.01.

**Figure 5:**
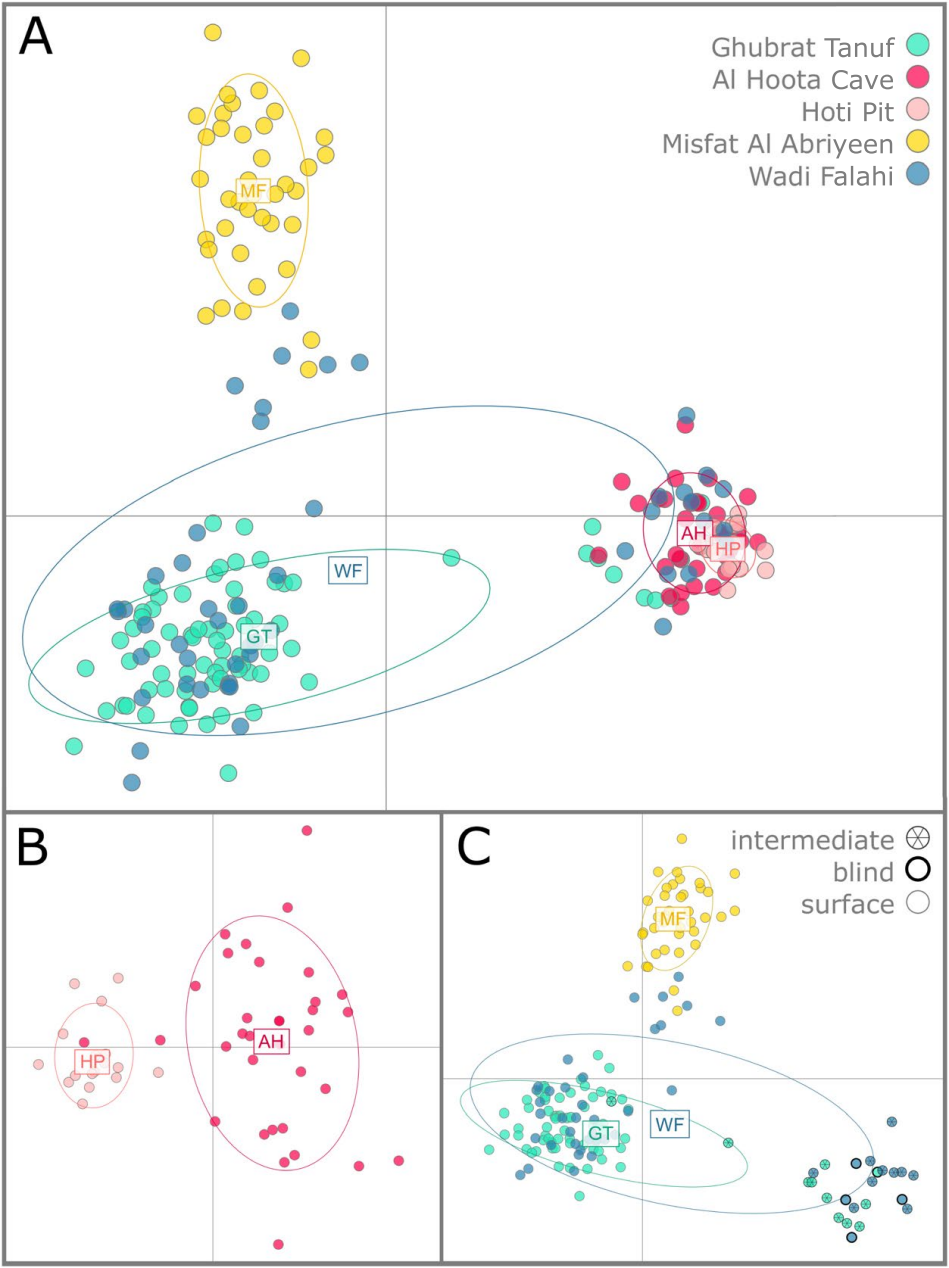
Results of PCA displaying the first two components of the analysis. A) PCA calculated for the whole data set, B) PCA calculated for the cave subset (Al Hoota and Hoti Pit), C) PCA calculated for the surface subset (Ghubrat Tanuf, Misfat and Wadi Falahi). The ellipses represent 95% of the inertia (variance) of this group. In C, specimens from surface sites exhibiting an intermediate phenotype are marked with an asterisk, blind individuals are marked with a black outline.

The results of the principal component analyses (PCA) display a tripartition roughly dividing the data set in a group comprising most of the Ghubrat Tanuf and Wadi Falahi samples, one cave group containing both Al Hoota and Hoti Pit samples and the third group including all samples from Misfat (Fig. 5A). In Fig. 5B, one can see a clear separation between samples of the two cave sites (as also depicted in Fig. 2). However, these separations between the surface samples are not so clear as some Wadi Falahi and Ghubrat Tanuf samples appear to be closer to the cave group than to the other surface clusters. This is due to individuals with an intermediate or blind phenotype from these localities (Fig. 5C). Regarding the samples of each locality, the Wadi Falahi population is by far the most variable one, with samples appearing in the diagram close to all other populations. This is also depicted by the corresponding inertia ellipses. The function reveals that ~24% of the information (variance) are explained by the first two components in the whole data set. Regarding the cave and surface subsets (Fig. 4B and C), the first two principal components explain ~19% of the variance in the cave subset and ~14% in the surface subset.

### Origin of individuals with intermediate phenotype

The presence of individuals with an intermediate phenotype raises questions whether this phenotype is the result of hybridisation or different environmental conditions (e.g. exposure to sunlight) on the species’ phenotypic variability. These intermediate phenotypes show the whole palette from normally developed eyes without pigmentation to pigmentation without visible eyes at all (Supplementary Figure S2). In most cases, these specimens have microphthalmic eyes and reduced pigmentation. After we found such specimens at Wadi Falahi and Ghubrat Tanuf, we intensified the search for intermediate individuals, which are only rarely found. We added all these individuals to our investigation. Consequently, the proportion between surface and intermediate specimens presented in this study (see below) does not reflect their actual occurrence in the population. However, single juvenile individuals bear eyes only one-sided or appear to be extraordinary pale or dark, respectively, considering the habitat in which they were caught (see also Fig. 5 in ref. 21). Since larvae of the cave morphotype also exhibit eyes when they hatch (which stop to develop and are overgrown by surrounding dermal tissue later in development), it is hard to differentiate whether these individuals would have developed into a troglomorphic or intermediate phenotype^19^. Hence, we chose a conservative approach and treated them as intermediate phenotypes (see phenotype assignment in Fig. 1).

Of the eleven intermediate, juvenile or blind individuals collected at Ghubrat Tanuf (among 62 normal surface individuals) one individual is truly troglomorphic (eyes only rudimentary, no pigmentation) with a 100% cave genotype, five individuals exhibit an intermediate phenotype (microphthalmic eyes and weak to no pigmentation) with 50% to 90% share of cave genotypes, and the remaining five individuals were juveniles with unusually small eyes and weak pigmentation and their genotypes exhibit 80% to 90% cave affiliation (Table 3, Supplementary Figure S2).

**Table 3:** List of individuals with intermediate phenotype/genotype collected at Ghubrat Tanuf.

Nr.	Individual ID	Phenotype	Eye development	Eye diameter (%)	Pigmentation	Genotype affiliation (%) cave/surface	Cave population affiliation (%) Al Hoota/Hoti Pit
1	Gbar221	blind	no eyes		none	100/0	100/0
2	Gbar390	intermed	microphthalmic	17.939	very pale	~ 50/50	~ 25/75
3	Gbar346	juv	microphthalmic	7.506	very pale	~ 80/20	0/100
4	Gbar355	intermed	microphthalmic	19.320	none	0/100	~ 20/80
5	Gbar356	intermed	microphthalmic	16.595	none	~ 90/10	0/100
6	Gbar357	intermed	microphthalmic	12.230	none	~ 90/10	0/100
7	Gbar358	intermed	microphthalmic	6.667	none	~ 80/20	0/100
8	Gbar359	juv	microphthalmic	12.879	very pale	~ 80/20	0/100
9	Gbar360	juv	microphthalmic	15.686	very pale	~ 80/20	0/100
10	Gbar361	juv	microphthalmic	14.779	very pale	~ 90/10	0/100
11	Gbar362	juv	microphthalmic	11.187	very pale	~ 90/10	~ 10/90

**Figure 6:**
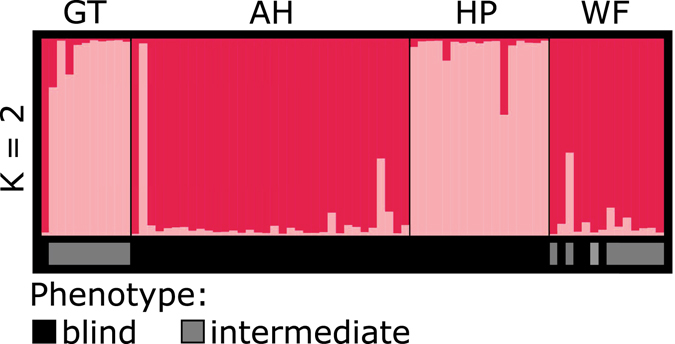
Population structure analysis based on the concatenated data set of cave individuals (AH and HP) and all blind or intermediate individuals collected from Ghubrat Tanuf or Wadi Falahi. The figure (K = 2) represents the two most probable clusters (magenta = Al Hoota and blind/intermediate individuals mostly from Wadi Falahi, light pink = Hoti Pit and blind/intermediate individuals mostly from Ghubrat Tanuf) resulting from the structure analysis. The corresponding phenotypes of the individuals are indicated by the bar below: black = blind, grey = intermediate.

Phenotype: blind = cave phenotype, intermed = intermediate phenotype, juv = juvenile individual. Eye development: no eyes = no eyes or eye structures externally visible, microphthalmic = small (underdeveloped) eyes visible. Eye diameter (in % of head length): if eyes were present, the eye diameter was measured relatively to head length (from snout tip to end of operculum of the specimen). Pigmentation: none = no sign of pigmentation visible, very pale = less pigmentation than normal surface phenotype. Genotype affiliation: assignment to the cave/surface cluster of each individual (%) according to the structure analysis (see Fig. 1). Cave population affiliation: assignment (%) to one of the cave populations (Al Hoota Cave or Hoti Pit) of each individual according to the structure analysis (see Fig. 6).

In Wadi Falahi 14 conspicuous individuals (among 36 normal surface individuals) were collected, five of which were completely troglomorphic without visible eyes and weak or no pigmentation, three were categorized as intermediate due to the presence of microphthalmic eyes and weak pigmentation and six individuals were still juvenile, but exhibited either microphthalmic eyes or no eyes at all and were only very weakly pigmented (Supplementary Figure S2). However, all of these 14 individuals possessed a 100% cave genotype (Table 4).

**Table 4:** List of individuals with intermediate phenotype/genotype collected at Wadi Falahi.

Nr.	Individual ID	Phenotype	Eye development	Eye diameter (%)	Pigmentation	Genotype affiliation (%) cave/surface	Cave population affiliation (%) Al Hoota/Hoti Pit
1	Gbar18	intermed	microphthalmic	9.401	very pale	100/0	100/0
2	Gbar491	blind	no eyes		very pale	100/0	100/0
3	Gbar538	intermed	microphthalmic	6.053	none	100/0	~ 60/40
4	Gbar539	blind	no eyes		none	100/0	100/0
5	Gbar540	blind	no eyes		very pale	100/0	100/0
6	Gbar541	intermed	microphthalmic	4.800	very pale	100/0	100/0
7	Gbar542	blind	no eyes		none	100/0	100/0
8	Gbar543	intermed	microphthalmic	8.491	none	100/0	~ 85/15
9	Gbar551	juv	microphthalmic	6.040	very pale	100/0	100/0
10	Gbar552	juv	microphthalmic	11.876	very pale	100/0	~ 90/10
11	Gbar553	juv	no eyes		very pale	100/0	100/0
12	Gbar554	juv	no eyes		very pale	100/0	100/0
13	Gbar555	juv	microphthalmic	10.824	very pale	100/0	100/0
14	Gbar556	juv	no eyes		very pale	100/0	100/0

Phenotype: blind = cave phenotype, intermed = intermediate phenotype, juv = juvenile individual. Eye development: no eyes = no eyes or eye structures externally visible, microphthalmic = small (underdeveloped) eyes visible. Eye diameter (in % of head length): if eyes were present, the eye diameter was measured relatively to head length (from snout tip to end of operculum of the specimen). Pigmentation: none = no sign of pigmentation visible, very pale = less pigmentation than normal surface phenotype. Genotype affiliation: assignment to the cave/surface cluster of each individual (%) according to the structure analysis (see Fig. 1). Cave population affiliation: percentage of genotype belonging to one of the cave populations (Al Hoota Cave or Hoti Pit) of each individual according to the structure analysis (see Fig. 6).

In order to explore the origin of these intermediate individuals, we ran a *structure* analysis comprising only cave individuals from Al Hoota and Hoti Pit and all individuals with a blind or intermediate phenotype (including also juveniles with conspicuous phenotype) from Ghubrat Tanuf and Wadi Falahi (Fig. 6). Again, the highest probability lies with K = 2 (ΔK) displaying that all specimen from Ghubrat Tanuf belong genetically to Hoti Pit (between 100% and 75% affiliation) except for one individual. This particular individual (Gbar221) is the only one from Ghubrat Tanuf exhibiting a true blind phenotype and belongs genetically 100% to the Al Hoota cluster. On the other hand, all individuals from Wadi Falahi belong genetically to the Al Hoota cluster (between 100% and 85% affiliation), whereas one specimen has a rather low (only a 60%) affiliation to the Al Hoota cluster (Gbar538). These findings are also supported by the results obtained from GeneClass2 analysis (Supplementary Table S5), where all intermediate individuals from Wadi Falahi were assigned to the Al Hoota population (one with high significance), and most intermediate individuals from Ghubrat Tanuf were assigned to the Hoti Pit population with the exception of Gbar221, which was assigned also to Al Hoota (highly significant).

**Figure 7:**
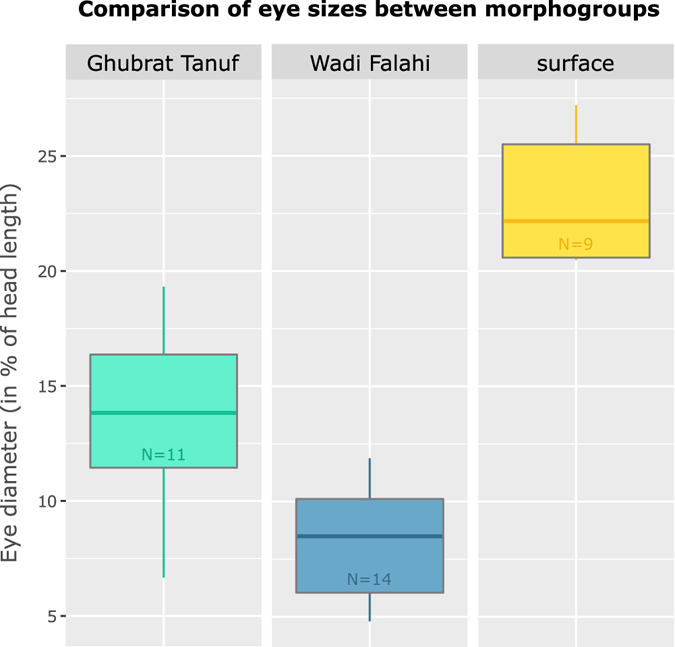
Boxplot based on eye sizes of intermediate individuals collected from Ghubrat Tanuf and Wadi Falahi compared to surface individuals with external fully developed eyes. The figure displays the difference in eye diameter between individuals with intermediate and surface phenotype. Y-axis represents the percentage of eye diameter measured relatively to head length. X-axis shows the three groups that were compared: intermediate individuals from Ghubrat Tanuf (N = 11, turquois), intermediate individuals from Wadi Falahi (N = 14, blue), and surface individuals (N = 9, three specimens from each surface site - Ghubrat Tanuf, Wadi Falahi and Misfat Al Abriyeen, yellow). Each box is confined by its first and third quartiles (contains 50% of data), the thick notch within the box represents the median (95% confidence interval) and the whiskers extend to the largest/smallest values of each population.

Concerning variation in eye sizes, the results of the comparative eye diameter measurements revealed a clearly smaller eye size in intermediate specimens compared to surface individuals with fully developed eyes (see Table 5). This becomes obvious in Fig. 7, where the smallest eyes were observed in intermediate individuals from Wadi Falahi (mean size 9% of head length) followed by slightly bigger eyes in intermediate individuals from Ghubrat Tanuf (mean size 14% of head length) and the biggest eyes in surface form (mean size 22% of head length).

**Table 5:** Eye diameter of selected surface individuals collected at Ghubrat Tanuf, Misfat Al Abriyeen and Wadi Falahi.

Nr.	Individual ID	Locality	Phenotype	Eye development	Eye diameter (%)	Pigmentation	Genotype affiliation (%) cave/surface
1	Gbar31	GT	surface	fully developed	20.46	present	0/100
2	Gbar67	GT	surface	fully developed	25.501	present	0/100
3	Gbar88	GT	surface	fully developed	24.386	present	0/100
4	Gbar280	MF	surface	fully developed	22.172	present	0/100
5	Gbar282	MF	surface	fully developed	27.199	present	0/100
6	Gbar286	MF	surface	fully developed	25.810	present	0/100
7	Gbar211	WF	surface	fully developed	20.494	present	0/100
8	Gbar212	WF	surface	fully developed	21.438	present	0/100
9	Gbar213	WF	surface	fully developed	20.582	present	0/100

All specimens bear a typical surface phenotype with fully developed eyes and pigmentation. Locality: GT = Ghubrat Tanuf, MF = Misfat Al Abriyeen, WF = Wadi Falahi. Eye diameter (in % of head length): eye diameter was measured relatively to head length (from snout tip to end of operculum of the specimen). Genotype affiliation: assignment to the cave/surface cluster of each individual (%) according to the structure analysis (see Fig. 1).

## Discussion

Concerning the genetic diversity within populations, the fact that several parameters like the number of alleles, frequency of polymorphic loci, genetic variability as well as the mean heterozygosity are all lower in the investigated cave populations than in surface populations (which is similar to findings in cave and surface populations of *Astyanax* ^8^) supports the hypothesis that the cave populations probably went through repeated genetic bottlenecks as suggested by Kruckenhauser *et al*.^21^. Moreover, it implies that the cave populations originated from a group of surface ancestors holding only a fraction of the genetic diversity of the whole species. Given the low genetic distance between cave and surface populations as reported by Kruckenhauser *et al*.^21^ who further suggested that the cave population of *G*. *barreimiae* is phylogenetically young, it seems plausible that this population originated at a time, when the climate started to change from wet to arid resulting in dropping water levels^23, 47, 48^. In southern Arabia the climate changed repeatedly in the Late Pleistocene, with two major wet periods, one of them coinciding with the last interglacial period (125 to 120 ka) and another during the early Holocene (9.7 to 6.2 ka)^48^. One of these changes probably disrupted the connection between surface and cave water bodies, leaving a subpopulation trapped inside the cave and isolated from surface water bodies, which also became more fragmented. However, it cannot be excluded that the cave population emerged from parallel speciation as found in *Astyanax* ^9^. Concerning the surface populations, H_e_ is higher than H_o_ in Ghubrat Tanuf and Wadi Falahi, whereas they are nearly similar in the other localities. Since the surface individuals from Ghubrat Tanuf, Wadi Falahi and Misfat all belong to the same cluster in the *structure* analyses (Fig. 1), it seems likely that gene flow between these sites occurs when waterways are easily traversable in season of high precipitations. This is also supported by the rather low fixation indices between the surface sites (0.021 to 0.083) suggesting increased gene flow compared to the fixation indices between both cave sites (0.093) and between surface and cave sites (0.101 to 0.244).

In the course of investigating potential gene flow and/or genetic differentiation between cave and surface populations, the *structure* analysis clearly shows that the troglomorphic individuals from Al Hoota Cave and Hoti Pit are differentiated from surface populations (except individuals with intermediate phenotype). Since not a single individual from one of the cave sites bears any degree of surface genotype, we conclude that there is no gene flow from the surface populations into the caves. The *structure* analysis of only the troglomorphic cave individuals revealed a differentiation between the individuals of Al Hoota Cave and Hoti Pit (Fig. 2), which became also apparent in the PCA (Fig. 5B). A possible explanation for the two individuals from Al Hoota cave bearing partial Hoti Pit genotype might be a temporal subterranean connection allowing unidirectional migration from Hoti Pit (located at higher altitude) to Al Hoota Cave during flood events. Apart from these two individuals, no sign for ongoing gene flow between both cave populations was detected, indicating that they are isolated from each other. As the individuals from Hoti Pit have a lower number of alleles which are mostly a subset of the Al Hoota alleles and have very few private alleles one can conclude that the Hoti Pit population originated from individuals of the Al Hoota Cave.

The differentiation of the Misfat population in the *structure* analysis with the second highest probability (K = 3, Fig. 1) from the other two surface populations is in accordance with the results of Kruckenhauser *et al*.^21^ who investigated genetic distances (mt data) of individuals from several surface populations and found no geographic pattern within this group. Yet, this differentiation is only weak suggesting occasional gene flow. Although there is no direct connection between these sites during dry periods, there might be a temporal water junction (probably underground) enabling single individuals from Misfat to migrate into the Wadi Falahi when the water level rises during flood events.

Regarding individuals with intermediate phenotypes, the origin of these specimens was of particular interest. Those collected from Ghubrat Tanuf mostly have a mixed cave/surface genotype indicating that there must be at least some degree of gene flow from cave populations. These findings are also corroborated by the results of the CR network which clearly shows that some of these individuals share the same haplotype with typical surface individuals (HT 1 and 6, see Fig. 4). The results of the *structure* analyses (Fig. 6) show that the intermediate individuals found in Ghubrat Tanuf have genetic similarity to the Hoti Pit population. Since the water body in the Hoti Pit cave is secluded and no visible surface connection leading to Ghubrat Tanuf is known, we assume that either single individuals of Hoti Pit somehow managed to migrate downstream through the Al Hoota Cave, the wadi and Ain Al Msalla to Ghubrat Tanuf during flood events or there exists at least a temporary underground connection between these sites. This is supported by the finding of a single blind individual with typical cave phenotype and genotype in Ghubrat Tanuf. It seems reasonable that offspring of mixed mating couples can survive under cave conditions in Ghubrat Tanuf while in surface waters chances of survival and reproduction are low.

However, the situation with specimens bearing an intermediate phenotype from Wadi Falahi is different, because they all have a “pure” cave genotype (see *structure* analyses in Fig. 1) of Al Hoota Cave. In fact, all of these individuals also belong to the “cave” haplogroup in the CR network (Fig. 4). Most probably, these specimens are not the product of hybridisation, but cave individuals that managed to survive after they were flushed out of the lower entry of Al Hoota Cave. The average amount of rainfall in Oman is low and normally, rainwater drain away into the ground fast. However, sporadic rainfalls in this region can be heavy and lead to flooding of wadis. Flooding of the Wadi Hoota channels the water directly into the upper entry of the Al Hoota Cave and causes a flood wave that flushes through the cave with a rate of 100 m^3^ per second^20^ connecting the cave with the surface water bodies. Anyhow, since not a single individual exhibiting any degree of surface genotype was found within the cave, we conclude that the strong flow rate during these flooding events prevent surface individuals from migrating into the cave. The intermediate phenotype of these “pure” cave individuals could be explained by phenotypic plasticity. One could assume that these individuals left the cave at an early developmental stage, when eyes were still visible. Subsequently, the increased exposure to sunlight leads to darker pigmentation and less reduced eyes than in cave specimens which developed in complete dark habitat. Banister^19^ observed similar effects of light exposure in *G*. *barreimiae*. He described development of melanin in the skin and particular regrowth of optic lobes in cave individuals kept under light exposure within short periods of time (after four months). Phenotypic plasticity seems a reasonable explanation considering that the troglomorphic phenotype is of rather recent origin^21^. Similar observations were conducted with individuals of the Micos cave population of *Astyanax*, which is considered to be phylogenetically young. Therefore, individuals of this population bear rudimentary eyes that are less reduced compared to phylogenetically old troglodyte populations (e.g. El Abra region) whereas pigmentation is almost as pronounced as in the surface form but increases even more after exposure to light^49, 50^. Effects of light exposure can also be observed in the Atlantic molly *Poecilia mexicana*, a species occurring in the Mexican Cueva del Azufre – a system comprising both surface as well as cave habitats. Interestingly, individuals of this species show a gradual decrease of eye-size from the entrance and the front chambers, which receive dim light through numerous skylights, to the innermost chamber of the cave. Interestingly, the differences in eye sizes seem to be related to different amounts of light received in the chambers^51^. Subsequently, to clarify and quantify the phenotypic variability as a consequence of light exposure during ontogeny in individuals of *G*. *barreimiae*, comparative breeding and rearing experiments under controlled conditions are inevitable.

The fact that we found not a single hybrid individual at Wadi Falahi leads to the conclusion that hybridisation between cave individuals and surface individuals from Wadi Falahi is either not occurring at all or none of the F1 generation survives and reaches reproductive stage in the surface environment. Probably, hybrid larvae, which are pale and blind or with debility of sight, are at a disadvantage and more prone to predation compared to surface larvae. In addition, the cannibalistic nature of *G*. *barreimiae* as mentioned in Timmermann and Plath^52^ might also decrease the chance of survival. In contrast, in Ghubrat Tanuf the two morphotypes hybridize, which indicates that the individuals with a surface phenotype are capable of breeding in the cave environment. This is in accordance with our observation in the Wadi Bani Khalid cave (Oman), in which typical surface forms of *G*. *barreimiae* occur frequently and seem to survive well in the cave habitat.

## Conclusion

Although our results showed significant genetic differentiation between the surface and cave form of *G*. *barreimiae*, we found that gene flow between the two morphotypes is still possible in cave environment but occurs only unidirectional from the cave to surface populations. Hence the integrity of the Al Hoota and Hoti Pit population with their peculiar phenotypes is currently not threatened by admixture with surface morphotypes. However, the restricted local occurrence in only two caves makes them vulnerable to extinction due to environmental impacts. Furthermore, there are indications for phenotypic plasticity, probably due to light exposure, in individuals collected from surface populations with an allele composition typical for cave specimens but exhibiting an intermediate phenotype. The finding of hybrid specimens and cave individuals outside the caves indicate that at least a temporary connection, probably underground, between Hoti Pit cave and surface water bodies must exist. These inferences are supported by results deduced from a combination of mitochondrial, nuclear and morphological data. Furthermore, we found a substructure between the investigated surface populations leading to the conclusion that gene flow between surface water bodies occurs sporadically.

## Electronic supplementary material

Supplementary Figures and Tables

Supplementary Table S1

Supplementary Table S5
