# Supplementary material for: Investigating gene flow between the blind cavefish Garra barreimiae and its conspecific surface populations
Source: Sci Rep. 2017 Jul 11;7:5130. doi: 10.1038/s41598-017-05194-3 (PMC5506003; doi:10.1038/s41598-017-05194-3)
Supplement: Supplementary file 1 — Supplementary Figures and Tables [file 41598_2017_5194_MOESM1_ESM.docx]

**Investigating gene flow between the blind cavefish *Garra barreimiae* and its conspecific surface populations**

*Sandra Kirchner, Helmut Sattmann, Elisabeth Haring, Lukas Plan, Reginald Victor, Luise Kruckenhauser

**Supplementary Figures and Tables**


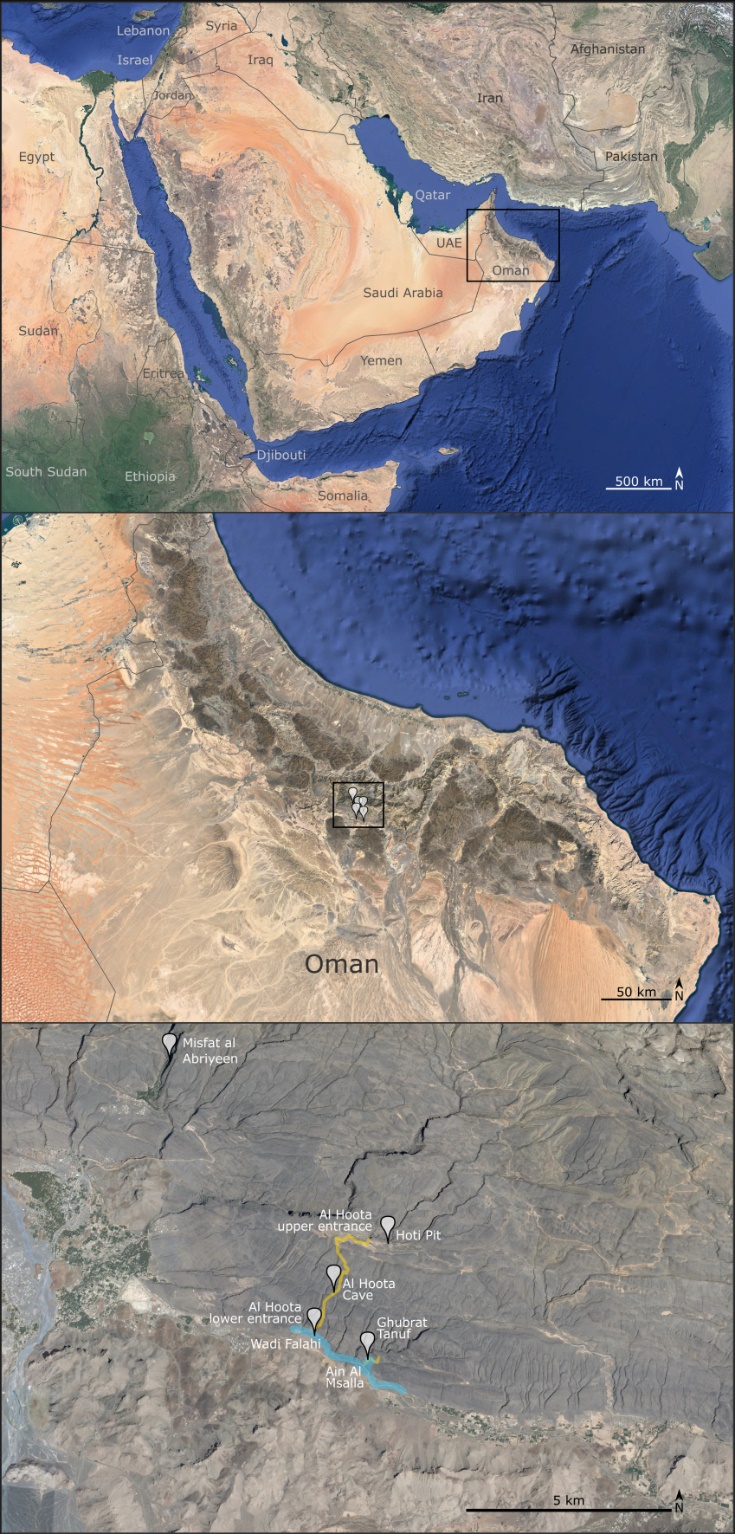


**Supplementary Figure S1. Sampling sites.** Geographic map of the Arabian Peninsula (top), detailed map of northern Oman displaying the sampling sites marked with a white flag (middle), more detailed map pointing out the five sampling sites in the midst of the Hajar Mountains (bottom), caves with underground water bodies are displayed with a yellow line, the wadi including surface water bodies is marked with a blue line. The map was created with the program TileMill version 0.10.1 (https://tilemill-project.github.io/tilemill/).


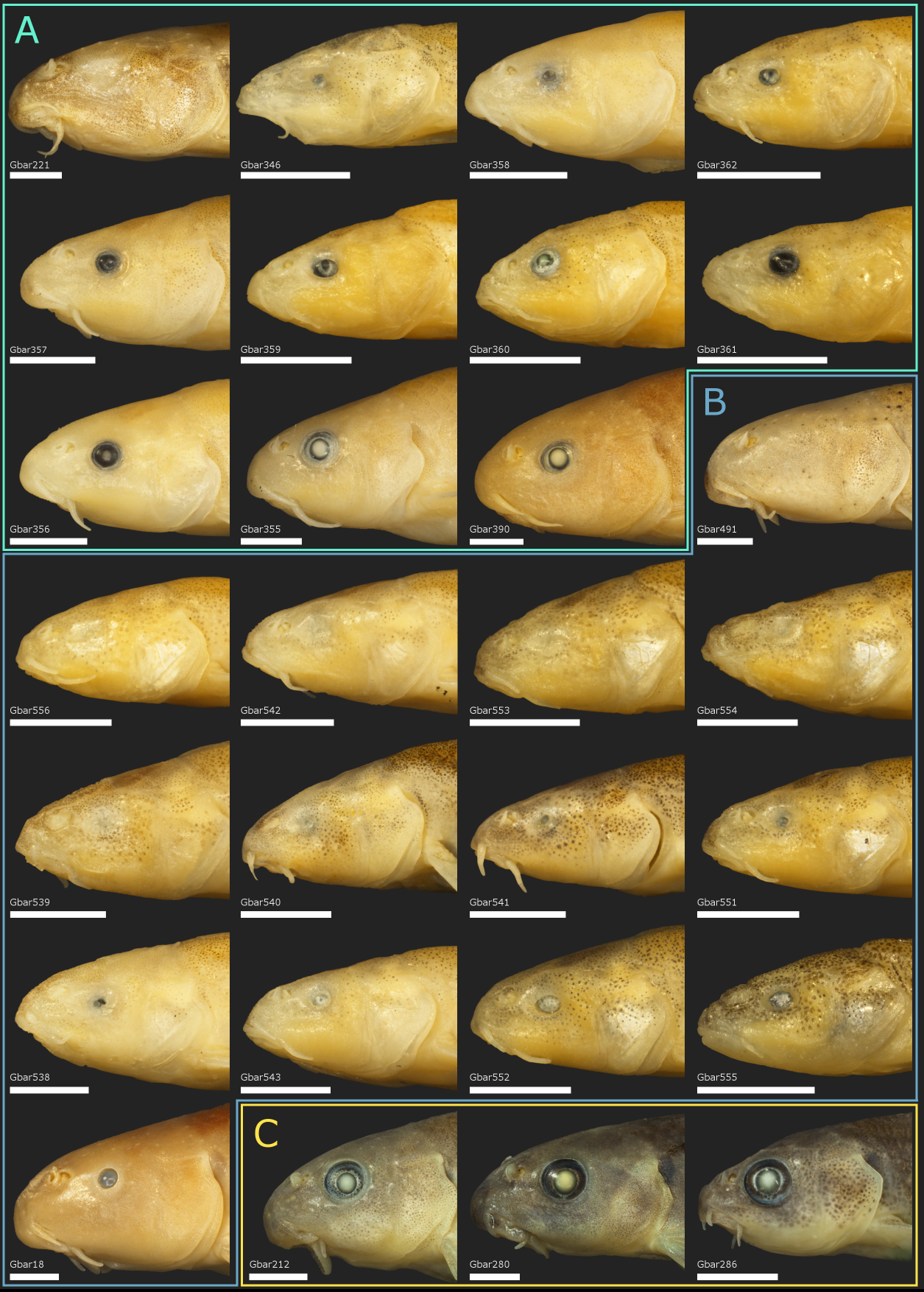


***Supplementary Figure S2. Depiction of varying eye development in intermediate and surface morphotypes.*** *Pictures of all intermediate/blind individuals collected from A) Ghubrat Tanuf (turquois) and B) Wadi Falahi (blue). C) Pictures of representatives of typical surface morphotype (yellow). The size of the white bar in each picture is correlated to a 2.5 mm scale.*

***Supplementary Table S1. Microsatellite data.***

*Table showing the genotypes of all 213 individuals in 18 loci. Label = individual identification code, population = code for sampling site (1 = Ghubrat Tanuf, 2 = Al Hoota Cave, 3 = Hoti Pit, 2 = Misfat al Abriyeen, 5 = Wadi Falahi), Phenotype = corresponding phenotype of each individual (surface, blind, intermediate, juvenile). Columns E to AM contain allele sizes of the corresponding microsatellite marker (2 columns per marker).*

**Supplementary Table S2. Sample information and population genetic parameters of the sampled morphotypes including/excluding individuals with intermediate phenotype.**

| Group | N | Na | A | Ar | pA | pA% | Ho | He | Fis | p | Ld | Lp% | HWE |
| --- | --- | --- | --- | --- | --- | --- | --- | --- | --- | --- | --- | --- | --- |
| **All individuals (N 213)** | | | | | | | | | | | | | |
| surface | 162 | 144 | 8.00 | 6.61 | 82 | 25.71 | 0.49 | 0.61 | 0.20 | 0.23 | PH8A, UHPE, JQSO, FYP9, JMLR, CJHG, 9XNC, 88CM, I6G2, 3ROZ | 100.00 | 0.00 |
| cave | 51 | 71 | 4.00 | 3.62 | 9 | 8.26 | 0.33 | 0.37 | 0.10 | 0.40 | UHPE, 9XNC | 83.33 | 0.005 |
| **Data set exclusive intermediate individuals (N 188)** | | | | | | | | | | | | | |
| surface | 137 | 138 | 7.67 | 6.41 | 82 | 59.42 | 0.51 | 0.59 | 0.14 | 0.23 | PH8A, UHPE, JQSO, FYP9, JMLR, CJHG, 9XNC, 88CM, I6G2, 3ROZ | 100.00 | 0.00 |
| cave | 51 | 71 | 3.94 | 3.63 | 9 | 12.68 | 0.33 | 0.37 | 0.10 | 0.40 | UHPE, 9XNC | 83.33 | 0.006 |

*The surface data set comprises: Ghubrat Tanuf, Misfat and Wadi Falahi; the cave data set comprises: Al Hoota Cave and Hoti Pit. N Number of analysed individuals per population, Na number of alleles per population, A mean number of alleles per locus, Ar allelic richness, pA number of private alleles, pA% percentage of private alleles per population (number of loci/population), H_o_ mean observed heterozygosity over all loci, H_e_ mean expected heterozygosity over all loci, Fis inbreeding coefficient, p (mean) p-value forHardy-Weinberg equilibrium (global test, mean over all loci), Ld Loci, that deviated from HW-equilibrium, Lp% percentage of polymorphic loci, HWE p-value for Hardy-Weinberg equilibria (global test).*

**Supplementary Table S3. Raw data of eye measurements.**

*List of eye and head measurements of individuals with intermediate/blind phenotype collected at Ghubrat Tanuf.*

| Nr. | Individual ID | Phenotype | Eye development | Eye diameter (mm) | Head length (mm) * | Eye diameter in % of head length |
| --- | --- | --- | --- | --- | --- | --- |
| 1 | Gbar221 | blind | no eyes |  |  |  |
| 2 | Gbar390 | intermediate | microphthalmic | 1.674 | 9.333 | 17.939 |
| 3 | Gbar346 | juvenile | microphthalmic | 0.351 | 4.672 | 7.506 |
| 4 | Gbar355 | intermediate | microphthalmic | 1.411 | 7.302 | 19.320 |
| 5 | Gbar356 | intermediate | microphthalmic | 1.086 | 6.544 | 16.595 |
| 6 | Gbar357 | intermediate | microphthalmic | 0.735 | 6.012 | 12.230 |
| 7 | Gbar358 | intermediate | microphthalmic | 0.363 | 5.442 | 6.667 |
| 8 | Gbar359 | juvenile | microphthalmic | 0.577 | 4.480 | 12.879 |
| 9 | Gbar360 | juvenile | microphthalmic | 0.677 | 4.317 | 15.686 |
| 10 | Gbar361 | juvenile | microphthalmic | 0.605 | 4.095 | 14.779 |
| 11 | Gbar362 | juvenile | microphthalmic | 0.464 | 4.146 | 11.187 |
| ** snout tip till end of operculum* | | |  |  |  |  |

*List of eye and head measurements of individuals with intermediate/blind phenotype collected at Wadi Falahi*

| Nr. | Individual ID | Phenotype | Eye development | Eye diameter (mm) | Head length (mm) * | Eye diameter in % of head length |
| --- | --- | --- | --- | --- | --- | --- |
| 1 | Gbar18 | intermediate | microphthalmic | 1.015 | 10.794 | 9.401 |
| 2 | Gbar491 | blind | no eyes |  |  |  |
| 3 | Gbar538 | intermediate | microphthalmic | 0.390 | 6.448 | 6.053 |
| 4 | Gbar539 | blind | no eyes |  |  |  |
| 5 | Gbar540 | blind | no eyes |  |  |  |
| 6 | Gbar541 | intermediate | microphthalmic | 0.238 | 4.960 | 4.800 |
| 7 | Gbar542 | blind | no eyes |  |  |  |
| 8 | Gbar543 | intermediate | microphthalmic | 0.458 | 5.396 | 8.491 |
| 9 | Gbar551 | juvenile | microphthalmic | 0.305 | 5.057 | 6.040 |
| 10 | Gbar552 | juvenile | microphthalmic | 0.607 | 5.108 | 11.876 |
| 11 | Gbar553 | juvenile | no eyes |  |  |  |
| 12 | Gbar554 | juvenile | no eyes |  |  |  |
| 13 | Gbar555 | juvenile | microphthalmic | 0.495 | 4.578 | 10.824 |
| 14 | Gbar556 | juvenile | no eyes |  |  |  |
| ** snout tip till end of operculum* | | |  |  |  |  |

*List of eye and head measurements of representative individuals with typical surface phenotype*

| Nr. | Individual ID | Locality | | Phenotype | Eye development | | Eye diameter (mm) | | Head length (mm) * | Eye diameter in % of head length |  |
| --- | --- | --- | --- | --- | --- | --- | --- | --- | --- | --- | --- |
| 1 | Gbar31 | Ghubrat Tanuf | | surface | fully developed | | 2.183 | | 10.667 | 20.467 |  |
| 2 | Gbar67 | Ghubrat Tanuf | | surface | fully developed | | 1.803 | | 7.069 | 25.501 |  |
| 3 | Gbar88 | Ghubrat Tanuf | | surface | fully developed | | 1.686 | | 6.912 | 24.386 |  |
| 4 | Gbar280 | Misfat Al Abriyeen | | surface | fully developed | | 2.240 | | 10.102 | 22.172 |  |
| 5 | Gbar282 | Misfat Al Abriyeen | | surface | fully developed | | 1.521 | | 5.594 | 27.199 |  |
| 6 | Gbar286 | Misfat Al Abriyeen | | surface | fully developed | | 1.488 | | 5.764 | 25.810 |  |
| 7 | Gbar211 | Wadi Falahi | | surface | fully developed | | 1.782 | | 8.693 | 20.494 |  |
| 8 | Gbar212 | Wadi Falahi | | surface | fully developed | | 1.804 | | 8.416 | 21.438 |  |
| 9 | Gbar213 | Wadi Falahi | | surface | fully developed | | 1.719 | | 8.354 | 20.582 |  |
| ** snout tip till end of operculum* | | |  | | |  |  |  | |  | |

Table containing measurements of eye diameter and head length (in mm) of intermediate/blind individuals from Ghubrat Tanuf and Wadi Falahi and representative individuals bearing a typical surface phenotype. The eye diameter was scaled to the corresponding head length (measured from snout tip till the end of the operculum) and is presented as percentage.

**Supplementary Table S4. List of haplotypes and corresponding individuals.**

| *218 total sequences (96 newly obtained sequences -> GenBank accession numbers MF183116-MF183211)* | | | | | |
| --- | --- | --- | --- | --- | --- |
| *122 sequences retrieved from Kruckenhauser et al., 2011* | | | | | |
| *MJ network based on 399 bp long fragment of CR* | | | | | |
| *Primers:* | *Thr1+Gbar* | | *GCATCGGTCTTGTAATCCGA* | | |
|  | *CR4-* | | *TTGGGCGTCGGCGGTGAGAG* | | |
| Haplotype | | IndID | Locality | Morphotype | Accession Nr. |
| 1 | | Gbar14 | Ghubrat Tanuf | surface |  |
| 1 | | Gbar15 | Ghubrat Tanuf | surface |  |
| 1 | | Gbar32 | Ghubrat Tanuf | surface |  |
| 1 | | Gbar33 | Ghubrat Tanuf | surface |  |
| 1 | | Gbar67 | Ghubrat Tanuf | surface |  |
| 1 | | Gbar90 | Ghubrat Tanuf | surface |  |
| 1 | | Gbar91 | Ghubrat Tanuf | surface |  |
| 1 | | Gbar94 | Ghubrat Tanuf | surface |  |
| 1 | | Gbar95 | Ghubrat Tanuf | surface |  |
| 1 | | Gbar332 | Ghubrat Tanuf | surface | MF183127 |
| 1 | | Gbar333 | Ghubrat Tanuf | surface | MF183128 |
| 1 | | Gbar335 | Ghubrat Tanuf | surface | MF183130 |
| 1 | | Gbar336 | Ghubrat Tanuf | surface | MF183131 |
| 1 | | Gbar337 | Ghubrat Tanuf | surface | MF183132 |
| 1 | | Gbar338 | Ghubrat Tanuf | surface | MF183133 |
| 1 | | Gbar340 | Ghubrat Tanuf | surface | MF183135 |
| 1 | | Gbar353 | Ghubrat Tanuf | surface | MF183146 |
| 1 | | Gbar354 | Ghubrat Tanuf | surface | MF183147 |
| 1 | | Gbar356 | Ghubrat Tanuf | intermediate | MF183149 |
| 1 | | Gbar357 | Ghubrat Tanuf | intermediate | MF183150 |
| 1 | | Gbar358 | Ghubrat Tanuf | intermediate | MF183151 |
| 1 | | Gbar360 | Ghubrat Tanuf | intermediate | MF183153 |
| 1 | | Gbar362 | Ghubrat Tanuf | intermediate | MF183155 |
| 1 | | Gbar372 | Ghubrat Tanuf | surface | MF183165 |
| 1 | | Gbar373 | Ghubrat Tanuf | surface | MF183166 |
| 1 | | Gbar387 | Ghubrat Tanuf | surface | MF183169 |
| 1 | | Gbar389 | Ghubrat Tanuf | surface | MF183171 |
| 1 | | Gbar392 | Ghubrat Tanuf | surface | MF183174 |
| 1 | | Gbar394 | Ghubrat Tanuf | surface | MF183176 |
| 1 | | Gbar395 | Ghubrat Tanuf | surface | MF183177 |
| 1 | | Gbar396 | Ghubrat Tanuf | surface | MF183178 |
| 1 | | Gbar397 | Ghubrat Tanuf | surface | MF183179 |
| 1 | | Gbar399 | Ghubrat Tanuf | surface | MF183181 |
| 1 | | Gbar417 | Ghubrat Tanuf | surface | MF183183 |
| 1 | | Gbar418 | Ghubrat Tanuf | surface | MF183184 |
| 1 | | Gbar419 | Ghubrat Tanuf | surface | MF183185 |
| 1 | | Gbar421 | Ghubrat Tanuf | surface | MF183187 |
| 1 | | Gbar49 | Wadi Falahi | surface |  |
| 1 | | Gbar54 | Wadi Falahi | surface |  |
| 1 | | Gbar55 | Wadi Falahi | surface |  |
| 1 | | Gbar132 | Wadi Falahi | surface |  |
| 1 | | Gbar133 | Wadi Falahi | surface |  |
| 1 | | Gbar135 | Wadi Falahi | surface |  |
| 1 | | Gbar136 | Wadi Falahi | surface |  |
| 1 | | Gbar138 | Wadi Falahi | surface |  |
| 1 | | Gbar139 | Wadi Falahi | surface |  |
| 1 | | Gbar140 | Wadi Falahi | surface |  |
| 1 | | Gbar141 | Wadi Falahi | surface |  |
| 1 | | Gbar211 | Wadi Falahi | surface |  |
| 1 | | Gbar212 | Wadi Falahi | surface |  |
| 1 | | Gbar213 | Wadi Falahi | surface |  |
| 1 | | Gbar214 | Wadi Falahi | surface |  |
| 1 | | Gbar215 | Wadi Falahi | surface |  |
| 1 | | Gbar216 | Wadi Falahi | surface |  |
| 1 | | Gbar488 | Wadi Falahi | surface | MF183198 |
| 1 | | Gbar492 | Wadi Falahi | surface | MF183200 |
| 2 | | Gbar221 | Ghubrat Tanuf | blind | MF183121 |
| 2 | | Gbar346 | Ghubrat Tanuf | intermediate | MF183141 |
| 2 | | Gbar359 | Ghubrat Tanuf | intermediate | MF183152 |
| 2 | | Gbar361 | Ghubrat Tanuf | intermediate | MF183154 |
| 2 | | Gbar8 | Al Hoota Cave | blind |  |
| 2 | | Gbar9 | Al Hoota Cave | blind |  |
| 2 | | Gbar10 | Al Hoota Cave | blind |  |
| 2 | | Gbar20 | Al Hoota Cave | blind |  |
| 2 | | Gbar21 | Al Hoota Cave | blind |  |
| 2 | | Gbar40 | Al Hoota Cave | blind |  |
| 2 | | Gbar42 | Al Hoota Cave | blind |  |
| 2 | | Gbar44 | Al Hoota Cave | blind |  |
| 2 | | Gbar80 | Al Hoota Cave | blind |  |
| 2 | | Gbar81 | Al Hoota Cave | blind |  |
| 2 | | Gbar82 | Al Hoota Cave | blind |  |
| 2 | | Gbar83 | Al Hoota Cave | blind |  |
| 2 | | Gbar85 | Al Hoota Cave | blind |  |
| 2 | | Gbar168 | Al Hoota Cave | blind | MF183116 |
| 2 | | Gbar169 | Al Hoota Cave | blind |  |
| 2 | | Gbar172 | Al Hoota Cave | blind |  |
| 2 | | Gbar173 | Al Hoota Cave | blind |  |
| 2 | | Gbar347 | Al Hoota Cave | blind | MF183142 |
| 2 | | Gbar349 | Al Hoota Cave | blind | MF183144 |
| 2 | | Gbar350 | Al Hoota Cave | blind | MF183145 |
| 2 | | Gbar474 | Al Hoota Cave | blind | MF183195 |
| 2 | | Gbar224 | Hoti Pit | blind | MF183124 |
| 2 | | Gbar225 | Hoti Pit | blind | MF183125 |
| 2 | | Gbar441 | Hoti Pit | blind | MF183189 |
| 2 | | Gbar444 | Hoti Pit | blind | MF183192 |
| 2 | | Gbar445 | Hoti Pit | blind | MF183193 |
| 2 | | Gbar446 | Hoti Pit | blind | MF183194 |
| 2 | | Gbar476 | Hoti Pit | blind | MF183197 |
| 2 | | Gbar510 | Hoti Pit | blind | MF183206 |
| 2 | | Gbar25 | Wadi Falahi | intermediate |  |
| 2 | | Gbar137 | Wadi Falahi | juvenile |  |
| 2 | | Gbar142 | Wadi Falahi | intermediate |  |
| 2 | | Gbar143 | Wadi Falahi | surface |  |
| 2 | | Gbar144 | Wadi Falahi | surface |  |
| 2 | | Gbar149 | Wadi Falahi | intermediate |  |
| 2 | | Gbar150 | Wadi Falahi | surface |  |
| 2 | | Gbar151 | Wadi Falahi | intermediate |  |
| 2 | | Gbar542 | Wadi Falahi | blind | MF183209 |
| 3 | | Gbar88 | Ghubrat Tanuf | surface |  |
| 3 | | Gbar180 | Al Hamra Falaj | surface |  |
| 3 | | Gbar181 | Al Hamra Falaj | surface |  |
| 3 | | Gbar191 | Misfat al Abriyeen | surface |  |
| 3 | | Gbar363 | Misfat al Abriyeen | surface | MF183156 |
| 3 | | Gbar365 | Misfat al Abriyeen | surface | MF183158 |
| 3 | | Gbar367 | Misfat al Abriyeen | surface | MF183160 |
| 3 | | Gbar369 | Misfat al Abriyeen | surface | MF183162 |
| 3 | | Gbar370 | Misfat al Abriyeen | surface | MF183163 |
| 3 | | Gbar47 | Wadi Falahi | surface |  |
| 3 | | Gbar112 | Wadi Falahi | surface |  |
| 3 | | Gbar131 | Wadi Falahi | surface |  |
| 3 | | Gbar154 | Wadi An Nakhar | surface |  |
| 3 | | Gbar156 | Wadi An Nakhar | surface |  |
| 3 | | Gbar157 | Wadi An Nakhar | surface |  |
| 3 | | Gbar160 | Wadi An Nakhar | surface |  |
| 3 | | Gbar162 | Wadi An Nakhar | surface |  |
| 3 | | Gbar175 | Wadi An Nakhar | surface |  |
| 4 | | Gbar184 | Al Hamra Falaj | surface |  |
| 4 | | Gbar505 | Hoti Pit | blind | MF183202 |
| 4 | | Gbar195 | Misfat al Abriyeen | surface |  |
| 4 | | Gbar196 | Misfat al Abriyeen | surface |  |
| 4 | | Gbar368 | Misfat al Abriyeen | surface | MF183161 |
| 4 | | Gbar198 | Wadi Dhum 1 | surface |  |
| 4 | | Gbar201 | Wadi Dhum 1 | surface |  |
| 4 | | Gbar203 | Wadi Dhum 1 | surface |  |
| 4 | | Gbar204 | Wadi Dhum 1 | surface |  |
| 4 | | Gbar205 | Wadi Dhum 1 | surface |  |
| 4 | | Gbar206 | Wadi Dhum 1 | surface |  |
| 4 | | Gbar207 | Wadi Dhum 1 | surface |  |
| 4 | | Gbar46 | Wadi Falahi | surface |  |
| 4 | | Gbar48 | Wadi Falahi | surface |  |
| 4 | | Gbar153 | Wadi An Nakhar | surface |  |
| 4 | | Gbar158 | Wadi An Nakhar | surface |  |
| 4 | | Gbar161 | Wadi An Nakhar | surface |  |
| 4 | | Gbar163 | Wadi An Nakhar | surface |  |
| 5 | | Gbar334 | Ghubrat Tanuf | surface | MF183129 |
| 5 | | Gbar339 | Ghubrat Tanuf | surface | MF183134 |
| 5 | | Gbar342 | Ghubrat Tanuf | surface | MF183137 |
| 5 | | Gbar374 | Ghubrat Tanuf | surface | MF183167 |
| 5 | | Gbar391 | Ghubrat Tanuf | surface | MF183173 |
| 5 | | Gbar393 | Ghubrat Tanuf | surface | MF183175 |
| 5 | | Gbar398 | Ghubrat Tanuf | surface | MF183180 |
| 5 | | Gbar400 | Ghubrat Tanuf | surface | MF183182 |
| 5 | | Gbar420 | Ghubrat Tanuf | surface | MF183186 |
| 5 | | Gbar134 | Wadi Falahi | surface |  |
| 6 | | Gbar92 | Ghubrat Tanuf | surface |  |
| 6 | | Gbar220 | Ghubrat Tanuf | surface | MF183120 |
| 6 | | Gbar222 | Ghubrat Tanuf | surface | MF183122 |
| 6 | | Gbar343 | Ghubrat Tanuf | surface | MF183138 |
| 6 | | Gbar344 | Ghubrat Tanuf | surface | MF183139 |
| 6 | | Gbar345 | Ghubrat Tanuf | surface | MF183140 |
| 6 | | Gbar355 | Ghubrat Tanuf | intermediate | MF183148 |
| 6 | | Gbar390 | Ghubrat Tanuf | intermediate | MF183172 |
| 7 | | Gbar192 | Misfat al Abriyeen | surface |  |
| 7 | | Gbar193 | Misfat al Abriyeen | surface |  |
| 7 | | Gbar194 | Misfat al Abriyeen | surface |  |
| 7 | | Gbar197 | Misfat al Abriyeen | surface |  |
| 7 | | Gbar218 | Misfat al Abriyeen | surface | MF183118 |
| 7 | | Gbar364 | Misfat al Abriyeen | surface | MF183157 |
| 7 | | Gbar366 | Misfat al Abriyeen | surface | MF183159 |
| 7 | | Gbar371 | Misfat al Abriyeen | surface | MF183164 |
| 8 | | Gbar43 | Al Hoota Cave | blind |  |
| 8 | | Gbar79 | Al Hoota Cave | blind |  |
| 8 | | Gbar84 | Al Hoota Cave | blind |  |
| 8 | | Gbar171 | Al Hoota Cave | blind |  |
| 8 | | Gbar217 | Al Hoota Cave | blind | MF183117 |
| 8 | | Gbar491 | Wadi Falahi | blind | MF183199 |
| 9 | | Gbar164 | Wadi Bani Habib | surface |  |
| 9 | | Gbar165 | Wadi Bani Habib | surface |  |
| 9 | | Gbar167 | Wadi Bani Habib | surface |  |
| 9 | | Gbar176 | Wadi Bani Habib | surface |  |
| 9 | | Gbar177 | Wadi Bani Habib | surface |  |
| 9 | | Gbar179 | Wadi Bani Habib | surface |  |
| 10 | | Gbar31 | Ghubrat Tanuf | surface |  |
| 10 | | Gbar87 | Ghubrat Tanuf | surface |  |
| 10 | | Gbar89 | Ghubrat Tanuf | surface |  |
| 10 | | Gbar376 | Ghubrat Tanuf | surface | MF183168 |
| 10 | | Gbar388 | Ghubrat Tanuf | surface | MF183170 |
| 10 | | Gbar146 | Wadi Falahi | surface |  |
| 11 | | Gbar442 | Hoti Pit | blind | MF183190 |
| 11 | | Gbar443 | Hoti Pit | blind | MF183191 |
| 11 | | Gbar506 | Hoti Pit | blind | MF183203 |
| 11 | | Gbar508 | Hoti Pit | blind | MF183205 |
| 12 | | Gbar11 | Al Hoota Cave | blind |  |
| 12 | | Gbar223 | Hoti Pit | blind | MF183123 |
| 13 | | Gbar22 | Al Hoota Cave | blind |  |
| 13 | | Gbar86 | Al Hoota Cave | blind |  |
| 14 | | Gbar539 | Wadi Falahi | blind | MF183208 |
| 14 | | Gbar556 | Wadi Falahi | blind | MF183211 |
| 15 | | Gbar114 | Wadi Falahi | surface |  |
| 15 | | Gbar174 | Wadi An Nakhar | surface |  |
| 16 | | Gbar182 | Al Hamra Falaj | surface |  |
| 16 | | Gbar504 | Hoti Pit | blind | MF183201 |
| 17 | | Gbar219 | Misfat al Abriyeen | surface | MF183119 |
| 17 | | Gbar226 | Misfat al Abriyeen | surface | MF183126 |
| 18 | | Gbar552 | Wadi Falahi | intermediate | MF183210 |
| 19 | | Gbar538 | Wadi Falahi | intermediate | MF183207 |
| 20 | | Gbar170 | Al Hoota Cave | cave |  |
| 21 | | Gbar440 | Hoti Pit | cave | MF183188 |
| 22 | | Gbar348 | Al Hoota Cave | cave | MF183143 |
| 23 | | Gbar475 | Al Hoota Cave | cave | MF183196 |
| 24 | | Gbar12 | Al Hoota Cave | cave |  |
| 25 | | Gbar507 | Hoti Pit | cave | MF183204 |
| 26 | | Gbar17 | Wadi Falahi | intermediate |  |
| 27 | | Gbar148 | Wadi Falahi | blind |  |
| 28 | | Gbar16 | Ghubrat Tanuf | surface |  |
| 29 | | Gbar113 | Wadi Falahi | surface |  |
| 30 | | Gbar129 | Wadi Falahi | surface |  |
| 31 | | Gbar50 | Wadi Falahi | surface |  |
| 32 | | Gbar183 | Al Hamra | surface |  |
| 33 | | Gbar13 | Ghubrat Tanuf | surface |  |
| 34 | | Gbar341 | Ghubrat Tanuf | surface | MF183136 |
| 35 | | Gbar202 | Wadi Dhum 1 | surface |  |
| 36 | | Gbar200 | Wadi Dhum 1 | surface |  |
| 37 | | Gbar199 | Wadi Dhum 1 | surface |  |
| 38 | | Gbar159 | Wadi An Nakhar | surface |  |
| 39 | | Gbar155 | Wadi An Nakhar | surface |  |
| 40 | | Gbar166 | Wadi Bani Habib | surface |  |
| 41 | | Gbar178 | Wadi Bani Habib | surface |  |

Table including information on haplotype, morphotype and sampling location of each individual used for the MJ network analysis of a 399 bp long fragment of the CR gene.
